# A novel HIV vaccine targeting the protease cleavage sites

**DOI:** 10.1186/s12981-017-0174-7

**Published:** 2017-09-12

**Authors:** Hongzhao Li, Robert W. Omange, Francis A. Plummer, Ma Luo

**Affiliations:** 1 Department of Medical Microbiology, University of Manitoba, Winnipeg, Canada; 2 National Microbiology Laboratory, Public Health Agency of Canada, Winnipeg, Canada; 3 JC Wilt Infectious Diseases Research Center, National Microbiology Laboratory, 745 Logan Avenue, Winnipeg, MB R3E 3L5 Canada

**Keywords:** HIV, Vaccine, Protease cleavage sites, Natural immunity, Pumwani sex worker cohort, Nonhuman primate, SIV

## Abstract

HIV preferentially infects activated CD4+ T cells and mutates rapidly. The classical vaccine approach aimed to generate broad immune responses to full HIV proteins largely failed to address the potential adverse impact of increased number of activated CD4+ T cells as viral targets. Learning from natural immunity observed in a group of HIV resistant Kenyan female sex workers, we are testing a novel vaccine approach. It focuses immune response to the highly conserved sequences surrounding the HIV protease cleavage sites (PCS) to disrupt viral maturation, while limiting excessive immune activation. Our pilot studies using nonhuman primate SIV infection models suggest that this approach is feasible and promising.

## Background

Worldwide, it is estimated that almost 37 million people are living with human immunodeficiency virus type 1 (HIV-1). In 2015 alone, around 2.1 million individuals became newly infected with HIV and 1.1 million people died from AIDS, highlighting the urgent need for an effective HIV vaccine. Six HIV vaccine candidates to date have been tested in Phase IIb clinical trials. The first two trials Vax004 and Vax003 sought to induce protection by eliciting antibody responses to gp120, but failed to protect against HIV acquisition [1, 2]. The three other phase IIb trials, HVTN502, 503 and 505, attempted to induce T cell-based immunity against HIV. All three failed to elicit immune responses capable of providing protection against HIV acquisition [3–5]. The only HIV vaccine that has been modestly successful was from the RV144 trial, in which a recombinant canary pox-based vaccine (ALVAC) combined with a recombinant gp120 (AIDSVAX) vaccine was tested. The vaccine protected 31% of vaccinees against HIV acquisition after a modified intention-to-treat analysis [6].

HIV primarily infects CD4+ T cells, a critical component of the human immune system. As a retrovirus HIV mutates rapidly, giving rise to extensive genetic diversity. These inherent characteristics underscore the challenges for developing a prophylactic vaccine. Novel approaches and ideas need to be tested to develop an effective vaccine to HIV-1.

## Natural immunity to HIV: a new clue to vaccine development

Edward Jenner developed the successful smallpox vaccine based on the natural immunity observed in milkmaids. Thus, the correlates of natural immunity to HIV-1 documented in highly exposed uninfected individuals may provide a vital clue for the development of a preventative vaccine for HIV-1. Several cohort studies have documented that there is considerable heterogeneity in susceptibility to HIV-1 infection [7–9]. Some individuals remain uninfected despite continued high risk exposure to HIV-1 [10]. Understanding why these individuals escape HIV-1 infection and the immunologic correlates that confer protective immunity in these individuals could aid in the development of an effective vaccine.

## The spectra of HIV Gag epitopes recognized by HLA alleles are associated with different outcomes of HIV-1 acquisition

Studies showed that the observed natural resistance to HIV-1 infection in the Pumwani sex worker cohort was associated with several alleles of human leukocyte antigens (HLAs) and specific CD8+ and CD4+ T cell responses to HIV-1 [11–14]. Therefore, we analyzed Gag epitopes of two major HIV-1 subtypes circulating in Kenya of two HLA class I alleles associated independently with different outcomes of HIV-1 infection. Our study showed that the protective allele, A*01:01, only recognized three Gag epitopes. In contrast, B*07:02, the allele associated with susceptibility, bound 30 epitope variants [15]. These two alleles differed most importantly in the spectrum of Gag epitopes they could present, and not in binding affinity, off-rates, the location of the epitopes, or epitope-specific Tem/Tcm frequencies [15]. Contrary to the classical HIV-1 vaccine design to generate broad and strong immune responses to several HIV-1 proteins [16, 17], the allele, which recognizes more epitopes and generates stronger IFN-γ ELISPOT responses, was associated with an increased susceptibility to HIV-1 acquisition.

## Lessons from natural immunity to HIV-1: more might not be better

Two things can be learned from our studies: (a) broad immune responses do not necessarily provide protection and may in fact promote infection; (b) narrowly targeted T cell response can be associated with protection against infection and may be an alternative strategy for an anti-HIV vaccine. Infection of CD4+ T cells is the key difference between HIV-1 and other infectious pathogens and activated CD4+ T cells are the primary targets for HIV-1, thus a narrow spectrum of epitope presentation by a protective allele appears to make sense. Theoretically, recognizing more epitopes will activate more CD8+ T cells to destroy the virally infected cells. However, it could also activate more bystander CD4+ T cells via cytokines produced by epitope-recognizing CD8+ T cells. The increased CD4+ T cell activation and recruitment to mucosal sites could increase the risk of HIV acquisition. This may explain why the B*07:02 allele, capable of recognizing a broader spectrum of Gag epitopes, was associated with rapid seroconversion. Ideally, an effective preventative vaccine to HIV-1 should be able to destroy the infecting virus or infected cells without causing excessive immune activation. The narrow and focused Gag epitope presentation by A*01:01 might provide such balance enabling the destruction of initially infected cells with minimum immune activation. Furthermore, recent studies have shown that in most cases mucosal acquisition of HIV-1 typically resulted from a single or a few founder viruses [18, 19]. Immune mechanisms preventing the establishment of a few founder viruses are likely different from the ones dealing with a full-blown viral infection after the virus has been well established in the host. It is possible that a lower magnitude, narrowly focused, well maintained virus-specific CD8+ T cell response to multiple subtypes is sufficient to destroy and eliminate a few founder viruses without inducing inflammatory responses that may activate more CD4+ T cells and provide more targets for HIV.

## Sequences around the HIV protease cleavage sites are viable vaccine targets

**Fig. 1:**
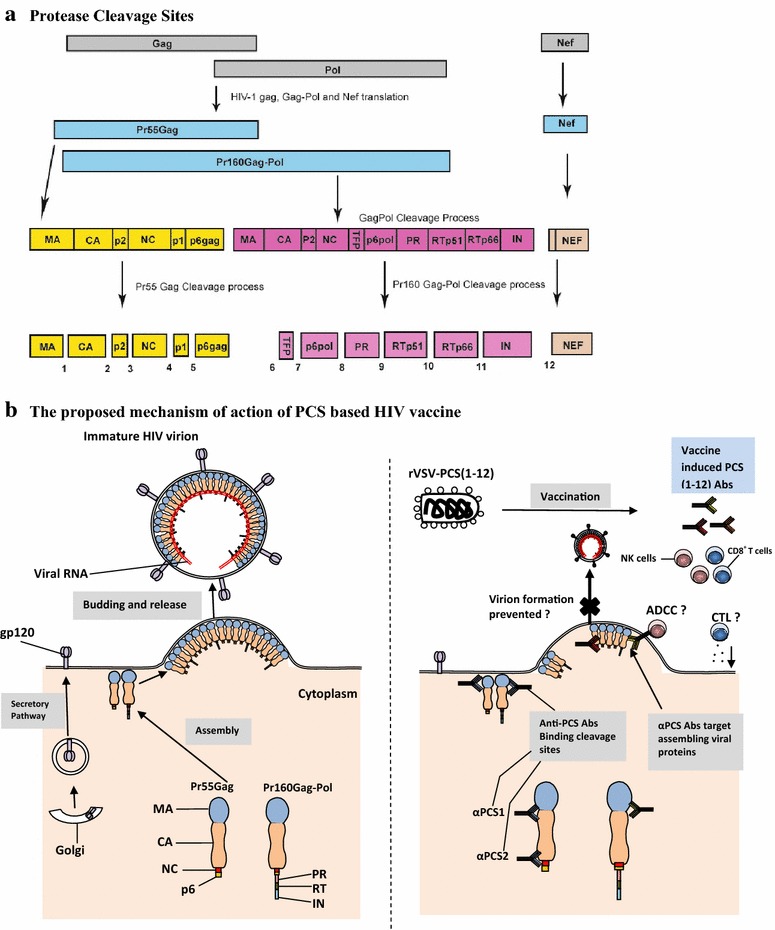
**a** Translation of HIV Gag and Pol genes produces two large polyproteins, Pr55Gag and Pr160Gag-Pol, which are cleaved at 12 protease cleavage sites (PCS) to produce 13 proteins in a mature HIV virion. Cleavage of Pr55Gag polyprotein by HIV protease at PCS1 produces matrix p17 (MA), at PCS2-capsid p24 (CA), PCS3-p2, PCS4-nucleocapsid/p1 (NC), PCS5-p1 and PCS6-p6^gag^. The cleavage of the Pr160 Gag-Pol polyprotein that is derived from ribosomal frame shifting results in the production of viral enzymes. Cleavage at PCS7 produces the transframe protein (TFP), at PCS8 produces p6^Pol^, PCS9-protease (PR), PCS10-reverse transcriptase (RT-p15), PCS11-RT-RNase H (RTp66)-integrase (IN) and PCS12-Nef [25–27]. **b** HIV envelope proteins gp120 and gp41 expression on the plasma membrane (PM) of infected cells occurs through secretory pathway involving the endoplasmic reticulum (ER), Golgi apparatus (GA) and membrane-bound vesicles. Transcription of HIV mRNA produces a precursor Gag polyprotein (Pr55Gag) containing HIV MA, CA, NC and p6 proteins. A precursor GagPol polyprotein (Pr160Gag-Pol) is synthesized by a frameshifting during transcription of Gag-encoding viral RNA and contains MA, CA, NC, PR, RT and IN domains. Viral assembly occurs on the inner surface of the PM, beginning with the binding of Gag on Pr55Gag and Pr160Gag-Pol to lipid rafts, and in the process Env (gp120 and gp41) is incorporated to the assembling complex [28]. The complex guides the budding leading to the formation of an immature virion, which eventually matures into an infectious virion after protease cleavage of Pr55Gag and Pr160Gag-Pol polyproteins incorporated in the virion. The PCS vaccine under evaluation uses 12 different recombinant vesicular stomatitis viruses (rVSV), each expressing a 20-amino acid peptide overlapping one of the 12 PCS. Vaccination with a combination of the 12 rVSVs in macaques elicits anti-PCS antibodies targeting different PCSs. The potential mechanism of the rVSV PCS vaccine may involve disrupting one or multiple stages of viral maturation and mediating cytotoxic killing of infected cells through antibody dependent cytotoxic cellular activity or cytotoxic T lymphocyte reactions

The only Gag peptide recognized by A*01:01 with relative high affinity and normal off-rate is a 9-mer peptide that covers the protease cleavage site at p17/p24 (Fig. 1a) [15]. This region is relatively conserved among major HIV subtypes (A1, B, D, and G). We tested 8 peptide variants of the HIV-1 subtype consensus sequences and found that A*01:01 can bind to all of them with similar affinity and off-rates [15]. Thus, despite A*01:01 being able to only present a narrow spectrum of Gag epitopes, it can tolerate variations of the specific epitope. Why is this region important for HIV-1? The protease of HIV-1 is a small 99-amino acid aspartic enzyme that mediates the cleavage of Gag, Gag-Pol and Nef precursor polyproteins (Fig. 1a). The process is highly specific, temporally regulated and essential for the production of infectious viral particles (Fig. 1b). Because a total of 12 proteolytic reactions are required to generate a viable virion, a vaccine generating immune responses to the sequences around the 12 protease cleavage sites of HIV-1 might be able to destroy virus-infected cells, drive viral mutations to generate non-infectious virus and take the advantage of the rapid mutations of HIV-1 (Fig. 1).

Because of its essential role in the production of infectious virions, HIV protease has been a major therapeutic target. Protease inhibitors have been successfully used to treat HIV-1 infection and are essential component of successful HAART therapies. Most of the protease inhibitors were designed to compete with its natural substrates based on the structure of the active binding site [20]. Recently, drugs that target Gag to inhibit protease-mediated processing at specific Gag cleavage sites have also been developed [21]. Studies have shown that the process of protease cleavage requires a tightly controlled, ordered sequence of proteolytic processing events mediated by different rates of cleavage at the different processing sites [22]. Even the subtle disturbances may be sufficient to interrupt this delicately balanced process and drive it toward a non-productive end [22]. Therefore, a vaccine targeting the 12 protease cleavage sites (PCS) could be effective. Furthermore, since the PCS are highly conserved among major subtypes of HIV-1, direct immune responses against these cleavage sites would yield several major advantages [23]. First, the host immune response could destroy the virus before it can establish itself permanently in the host. Second, the vaccine could force the virus to mutate, thus eliminating viable virions by abolishing the normal function of the HIV protease. Third, restricting the immune responses to these sites can avoid distracting immune responses that often generate unwanted inflammatory responses and excessive immune activation leading to more targets for HIV-1 infection, establishment and spread. A vaccine focusing on the sequences around the 12 PCS of HIV-1 is like a surgical attack of the function of HIV protease with 12 bullets, in the meantime minimizing the level of mucosal T cell activation, which has been proposed as a critical factor in developing an effective mucosal AIDS vaccine [24]. Since all 12 protease cleavage reactions have to be carried out successfully to generate an infectious virus, vaccines generating immune responses against the 12 substrates of HIV-1 protease could make it more difficult for the virus to escape in the meantime avoiding unfavorable effect.

## Evaluation of a PCS-targeting vaccine in nonhuman primates

Nonhuman primates (NHP) are the best animal models to evaluate candidate vaccines for human pathogens. PCS peptides delivered by recombinant vesicular stomatitis virus and nanoparticles (PCS vaccine) were tested in a pilot study as a preventative vaccine candidate using a cynomolgus macaque SIV infection model. Based on promising results from this pilot study, the vaccine is currently being validated and further characterized in a larger-scale study, in comparison with vaccines against full Gag and Env proteins.

## Conclusion

Based on the correlates of natural immunity to HIV resistance and our preliminary data from nonhuman primate study, the new vaccine strategy targeting the viral protease cleavage sites is feasible and promising. It needs to be further studied and refined towards the development of an effective HIV vaccine.
